# Supplementary material for: A large-scale method to measure the absolute stoichiometries of protein Poly-ADP-Ribosylation
Source: bioRxiv. 2025 Mar 27:2025.03.27.645734. Preprint. [Version 1] doi: 10.1101/2025.03.27.645734 (PMC11974908; doi:10.1101/2025.03.27.645734)
Supplement: 3 [file NIHPP2025.03.27.645734v1-supplement-3.pdf]

bioRxiv preprint doi: https://doi.org/10.1101/2025.03.27.645734; this version posted March 27, 2025. The copyright holder for this preprint (which was not certified by peer review) is the author/funder, who has granted bioRxiv a license to display the preprint in perpetuity. It is made available under aCC-BY 4.0 International license.

Supplementary Fig. 1

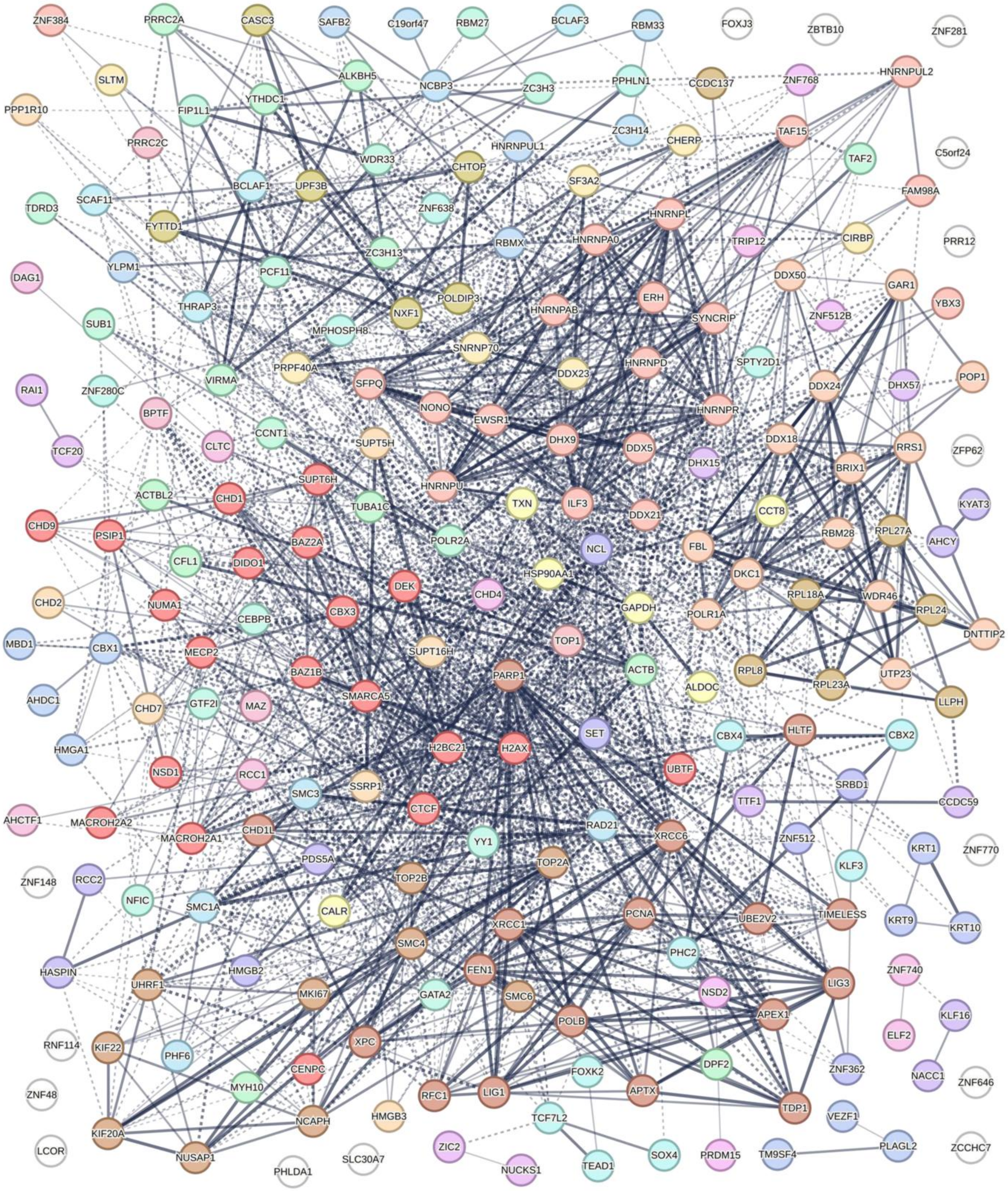

Figure S1. Functional connections among the PARylated proteins.

bioRxiv preprint doi: https://doi.org/10.1101/2025.03.27.645734; this version posted March 27, 2025. The copyright holder for this preprint (which was not certified by peer review) is the author/funder, who has granted bioRxiv a license to display the preprint in perpetuity. It is made available under aCC-BY 4.0 International license.

The network of functional connections among the 235 PARylated proteins identified in PARylation stoichiometry analysis was determined by STRING analysis (STRING Version 11.5).
